# Delineating the HMGB1 and HMGB2 interactome in prostate and ovary epithelial cells and its relationship with cancer

**DOI:** 10.18632/oncotarget.24887

**Published:** 2018-04-10

**Authors:** Aida Barreiro-Alonso, Mónica Lamas-Maceiras, Rosa García-Díaz, Esther Rodríguez-Belmonte, Lu Yu, Mercedes Pardo, Jyoti S. Choudhary, María Esperanza Cerdán

**Affiliations:** ^1^ EXPRELA Group, Centro de Investigacións Científicas Avanzadas, Departamento de Biología, Facultade de Ciencias, INIBIC-Universidade da Coruña, Campus de A Coruña, A Coruña, 15071, Spain; ^2^ Proteomic Mass Spectrometry, Wellcome Trust Sanger Institute, Wellcome Genome Campus, Hinxton, Cambridge, CB10 1SA, United Kingdom; ^3^ Present Address: The Institute of Cancer Research, Chester Beatty Laboratories, London, SW3 6JB, United Kingdom

**Keywords:** epithelial cells, high mobility proteins, protein-protein interactions, HMGB1, HMGB2

## Abstract

High Mobility Group B (HMGB) proteins are involved in cancer progression and in cellular responses to platinum compounds used in the chemotherapy of prostate and ovary cancer. Here we use affinity purification coupled to mass spectrometry (MS) and yeast two-hybrid (Y2H) screening to carry out an exhaustive study of HMGB1 and HMGB2 protein interactions in the context of prostate and ovary epithelia. We present a proteomic study of HMGB1 partners based on immunoprecipitation of HMGB1 from a non-cancerous prostate epithelial cell line. In addition, HMGB1 and HMGB2 were used as baits in yeast two-hybrid screening of libraries from prostate and ovary epithelial cell lines as well as from healthy ovary tissue. HMGB1 interacts with many nuclear proteins that control gene expression, but also with proteins that form part of the cytoskeleton, cell-adhesion structures and others involved in intracellular protein translocation, cellular migration, secretion, apoptosis and cell survival. HMGB2 interacts with proteins involved in apoptosis, cell motility and cellular proliferation. High confidence interactors, based on repeated identification in different cell types or in both MS and Y2H approaches, are discussed in relation to cancer. This study represents a useful resource for detailed investigation of the role of HMGB1 in cancer of epithelial origins, as well as potential alternative avenues of therapeutic intervention.

## INTRODUCTION

Carcinomas are the most frequent types of cancer, in which epithelial cells lose their normal cell-to-cell contacts, adopt a mesenchymal morphology and experiment a transformation to tumor cells [1]. Adenocarcinomas are epithelial cancers arising in glandular tissues and they comprise the largest group of human epithelial malignancies that develop in different organs.

HMGB1 and HMGB2 are members of the High Mobility Group (HMG) protein superfamily that contain a DNA binding domain (HMG-Box), and their overexpression has been associated to main cancer hallmarks, tumor progression, metastasis formation and bad prognosis [2]. The HMGB proteins are localized in the nucleus, cytoplasm and are also secreted to the extracellular milieu following either active secretion by immune cells or passive release from necrotic cells [3]. HMGB proteins are functionally related to cancer progression [4–7] and overexpression of the *HMGB1* gene has been detected in cancerous cells from epithelial origin in prostate and ovary [7, 8]. In addition, HMGB proteins have increased affinity for platinated DNA [9]. The HMG box-A of HMGB1 specifically recognizes the distorted GG pairs in the major adducts formed with cisplatin. The Phe37 in box-A stacks with Pt-GG and this enhances its DNA affinity [10]. The biological effect of HMGB1 binding to these adducts remains controversial and has been reported to facilitate DNA repair and cause a shielding effect towards NER factors [2, 11]. HMGB proteins are also involved in sensitizing cells to platinum compounds used in the chemotherapy of prostate and ovary cancer [12, 13]. Antagonists against HMGB1 have therapeutic use in lung cancer [14, 15] and restore sensitivity towards platinated compounds used in chemotherapy [16].

Protein interactions are ultimately responsible of cellular signaling and transformation; for this reason the identification of protein-protein interactions in ovarian and prostatic epithelial cells is crucial for the understanding of carcinoma origin and evolution in these organs. In the present work we describe proteins interacting with human HMGB1 and HMGB2 in ovarian and prostatic epithelial cells. Prostate PNT2 cells were used in proteomic studies based on immunoprecipitation of HMGB1 and identification of co-immunoprecipitating proteins by mass spectrometry. Libraries constructed with RNA from epithelial cell lines from prostate and ovary as well as from healthy ovary tissue were used to perform yeast two-hybrid screening using HMGB1 and HMGB2 baits. The nature and functions of the identified binding partners were analyzed and correlated to cancerous processes.

## RESULTS AND DISCUSSION

### Identification of HMGB1 interacting proteins in prostate epithelial cells by co-immunoprecipitation and mass spectrometry

**Figure 1:**
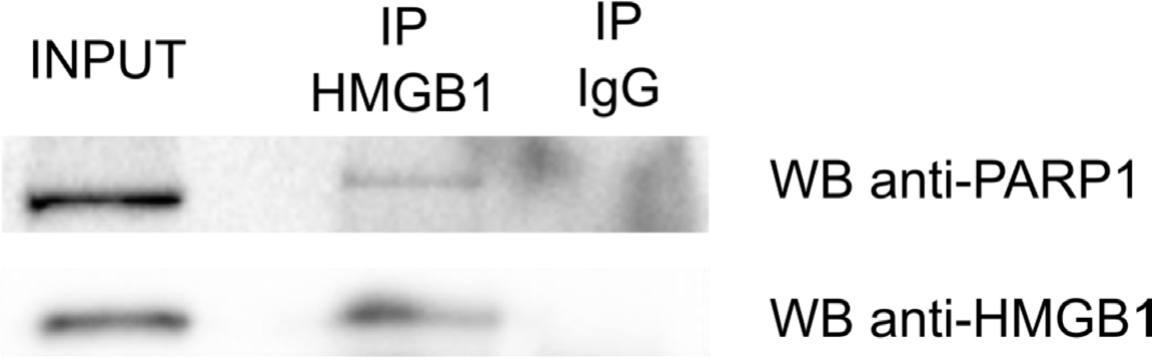
Co-Immunoprecipitation of HMGB1 and PARP1 in PTN2 cells

To identify proteins interacting with HMGB1, cell lysates from PNT2 prostate epithelial cells were incubated with anti-HMGB1 antibody or anti-rabbit IgG antibody (negative control) and immunoprecipitated proteins were identified by mass spectrometry as described in the materials and methods section. Two replicate experiments were performed. HMGB1 was specifically detected in the experimental replicates and not in the negative controls (Supplementary Table 1). We used SAINT analysis [17, 18] to derive a list of statistically significant true interactors. Preys with SAINT probability score cut-off of 1 detected by at least two unique peptides were deemed high confidence HMGB1 interacting proteins. The complete list of 159 proteins fulfilling these criteria is shown in Supplementary Table 1. To explore if any of the identified HMGB1 associated proteins was already known to interact with it, the list was matched against published HMGB1 binding partners in the BioGRID database [19]. There are 89 of such proteins annotated in the database, including HMGB1, since it has been reported to form dimmers and tetramers [20]. Six proteins from our list were annotated as HMGB1 interactors, validating our experimental approach: SUPT16H and SSRP1, the two subunits of the FACT complex, a general chromatin remodeler that reorganizes nucleosomes; PARP1, the poly(ADP-ribose) polymerase 1; the H3 Family 3A histone; HNRNPK, the heterogeneous nuclear ribonucleoprotein K, and heat shock protein HSPA8. We also confirmed the interaction of HMGB1 and PARP1 in PNT2 cells by co-immunoprecipitation (Figure 1).

**Figure 2:**
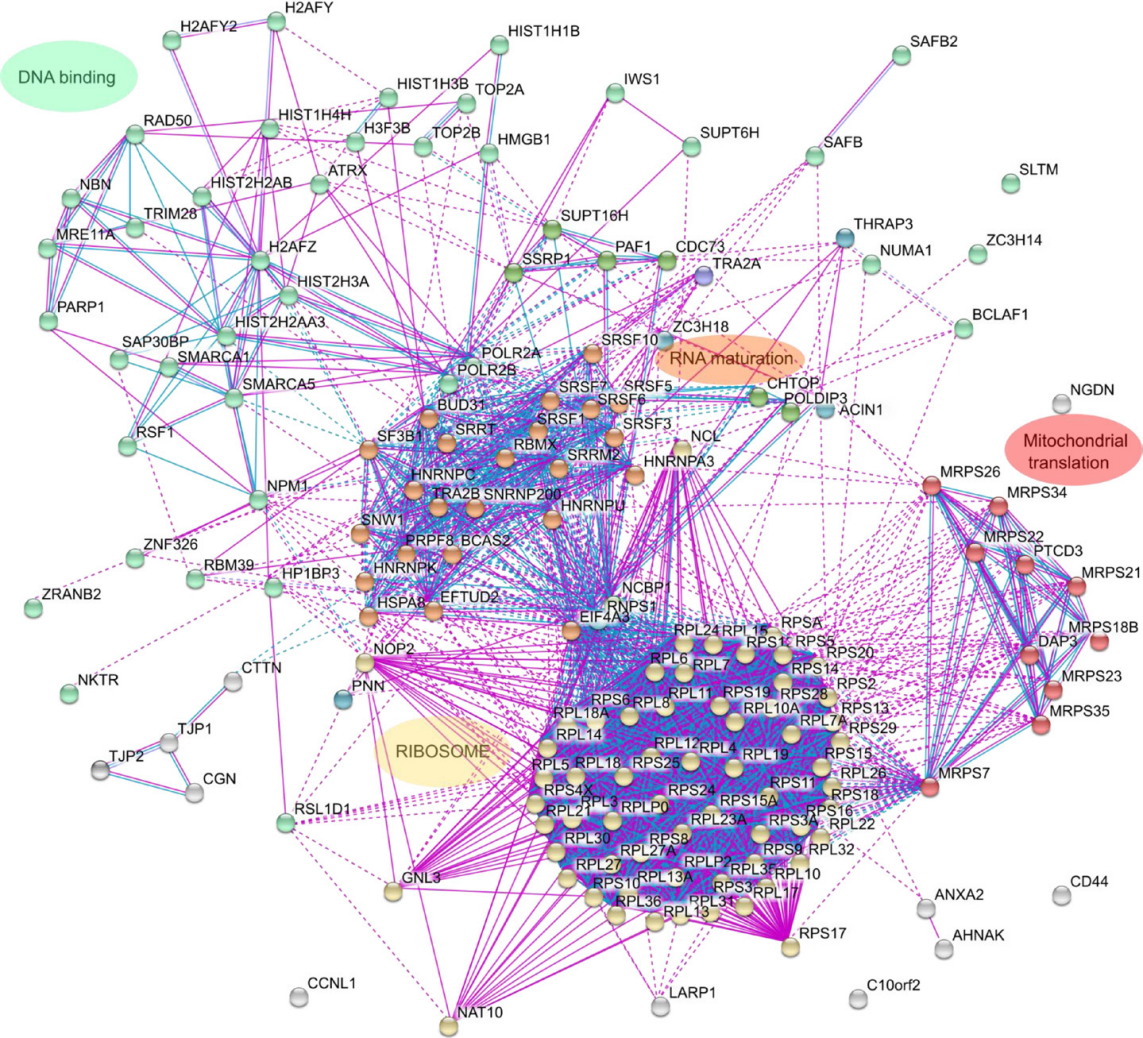
Clustering of proteins that interact with HMGB1 in PNT2 cells The interactome of HMGB1 partners, reflecting only experimental or data-base recorded interactions, has been constructed with STRING.

We then analyzed the list of HMGB1 interacting proteins in detail to explore their functional significance. We first looked for functional protein association networks amongst the identified HMGB1 preys with STRING [21]. The majority of proteins clustered into 4 clearly differentiated groups (Figure 2). Cluster 1 included DNA binding proteins involved in DNA transcription or DNA repair and proteins involved in chromatin structure and remodeling. Cluster 2 included proteins from the spliceosome. Cluster 3 comprised ribosomal proteins and/or proteins related to ribosome biogenesis. Cluster 4 proteins were implicated in mitochondrial translation. The role of HMGB1 in the regulation of gene expression has been previously reported and is mediated by diverse mechanisms: modulation of DNA template accessibility [22, 23], interaction with the general transcriptional machinery [24, 25] or control of the activity of specific transcriptional factors [23, 26]. In the nucleus HMGB1 binds DNA, which facilitates chromatin bending, an event required for its interaction with proteins engaged in transcription and DNA repair [2]. Our interaction proteomic study suggests that HMGB1 might also participate in other co or post-transcriptional events that affect gene expression. These results hint that HMGB1 interactions with other proteins might control gene expression in a very complex way at different stages, from regulating DNA template availability for transcription, through RNA synthesis, maturation and export of RNA from the nucleus to the cytoplasm and protein synthesis. In addition, coordination of mitochondrial translation with ribosomal protein synthesis is important in terms of bioenergetic balance; HMGB1 regulates mitochondrial bioenergetics in tumor cells [27] and HMGB1 binding proteins included in cluster 4 confirm this function.

Strikingly, in addition to proteins regulating gene expression, we also identified tight junction proteins TJP1 and TJP2 among HMGB1 interacting proteins. This is remarkable in light of HMGB1’s role in metastasis [14, 28] and points to potential players cooperating with HMGB1 to promote metastasis.

**Figure 3:**
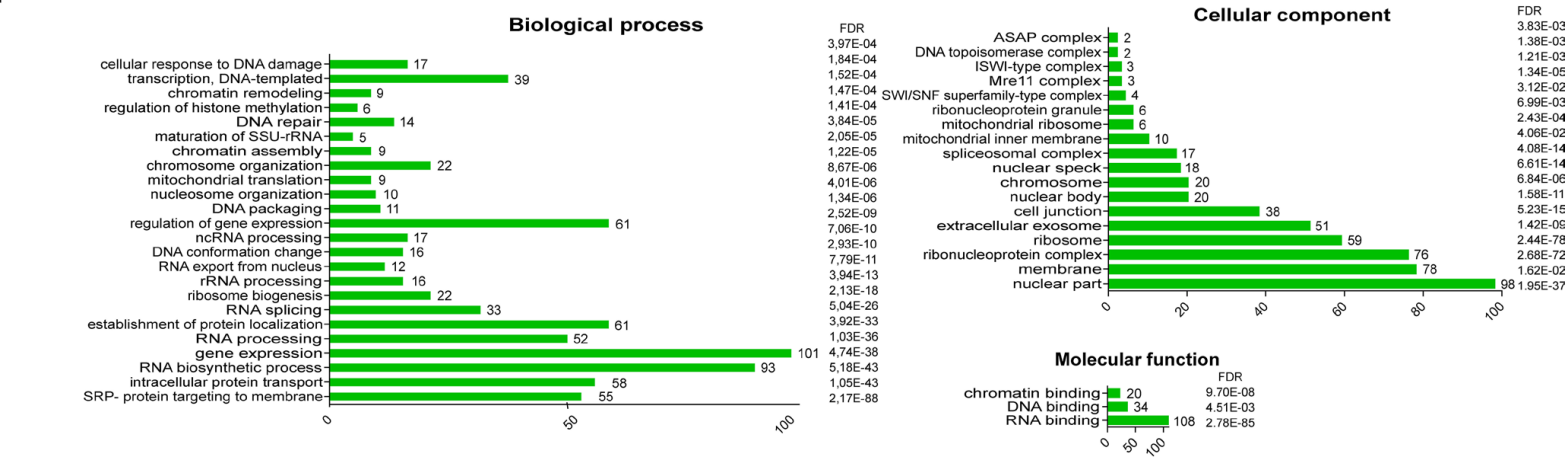
Go Term enrichment analysis of proteins that interact with HMGB1 in PNT2 cells

We next performed GO term enrichment analysis to functionally characterize the list (Figure 3). This showed that the clusters above coincided with statistically overrepresented terms. Regarding biological processes, enriched terms included “Gene expression” and “Regulation of gene expression”. Other overrepresented GO terms included chromatin/DNA modification, response to DNA damage, transcription mediated by RNA polymerase II, and RNA polymerase I, RNA export from nucleus, processing of mRNA, rRNA and ncRNA, and ribosome biogenesis. The GO term “SRP- dependent cotranslational protein targeting to membrane”, associated with 55 proteins, refers to the intracellular protein transport of ribosomes and nascent polypeptides to the ER membrane. These findings suggest a cytoplasmic function of HMGB1 in the control of ribosome biogenesis and protein synthesis, which could affect the proliferative capacity of cells. In support of this function of HMGB1 in the control of cell proliferation, it has been reported that ethyl pyruvate, a potent inhibitor of HMGB1 transport from nucleus to cytoplasm, greatly reduces cancerous cell proliferation [29, 30].

GO cellular component enriched terms included “nuclear part” and “ribosome”, in agreement with GO biological process terms “gene expression” and “ribosome biogenesis”. In addition, the term “extracellular exosome” was also over-represented. The 51 proteins annotated with this term included many ribosomal proteins. Extracellular vesicles are promising blood biomarkers since their cargoes may reflect the pathophysiological state in the cell of origin. The production of extracellular vesicles has been experimentally proven in PNT2 cells, and their mRNA content found to differ from that in exosomes from cancerous cells of epithelial origin [31]. HMGB1 itself was recently detected in epithelial cells exosomes [32].

### Identification of HMGB1 and HMGB2 binding proteins by yeast two-hybrid screening of cDNA libraries from human prostate and ovary epithelial cells

The identification of HMGB1 associated proteins based on co-immunoprecipitation and MS analysis was carried out in the non-cancerous, immortalized prostate epithelial cell line PNT2 containing a SV40 genome with a defective replication origin. To identify interactions in non-transformed cells, we then extended our analysis to human epithelial cells from prostate and ovary primary cultures (HPEpiC and HOSEpiC respectively). We generated cDNA libraries from RNA extracted from epithelial cells from prostate (HPEpiC) and ovary (HOSEpiC), and screened for interactions using HMGB1 and HMGB2 baits, as described in the Materials and methods section. We did not achieve saturation in the screen and there are probably other preys of HMGB1 and HMGB2 that were not captured.

### HMGB1 binding proteins in HPEpiC and HOSEpic cells

We identified 16 proteins as HMGB1 preys (Table 1) which can be classified in several functional groups: a) components of the cytoskeletal system: KRT7, and NEXN; b) cellular signaling in response to growth factors: ENAH, RPS20 and SPINT1 c) components of the cellular membrane and extracellular matrix and regulators of cell invasion and migration in tumoral cells: PCOLCE, HSPA5 UBE21, RPL29, CTNNBL1 and ZNF428; d) apoptosis-related proteins: EIF1, CCAR1 and ACBD3. None of these have previously been reported as HMGB1 interactors.

**Table 1:** HMGB1 preys in HOSEpic and HPEpiC cells

Gene	*N*	Cells	Function	Relation to cancer
*ACBD3*	7	HOSEpiC	Apoptosis	Golgi resident protein GCP60 (GOCAP1) can interact with a golgin-160 fragment to regulate cell apoptosis [58].
*C1QBP*	2	HOSEpiC	Cell motility	C1qBP protects the cells against staurosporine induced apoptosis and increases proliferation and cell migration in cancerous cells [59]. Predictor of tumor size in progesterone positive tumors and marker for proliferation in breast cancer [60].
*CCAR1*	3	HOSEpiC	Apoptosis	Cell division cycle and apoptosis regulator protein 1 (CARP-1) regulates cell growth and apoptosis by serving as a co-activator of several genes including steroid nuclear receptors and tumour suppressor p53 [49].
*CTNNBL1*	1	HOSEpiC	Tumour progression	The spliceosome-associated factor, β-catenin-like protein 1, is overexpressed in high-grade serous ovarian carcinomas. Moreover, this protein can promote proliferation and invasion in ovarian cancer cells [29].
*EIF1/SUI1*	1	HPEpiC	Apoptosis	Eukaryotic translation initiation factor 1 (eIF1) is activated by oncogene *MCT-1* [61].
*ENAH*	2	HPEpiC	Cellular signalling	Protein enabled homolog. Involved in PI3K-dependent cell invasion induced by Platelet-derived growth factor in human breast cancer cells [62]. Involved in human lung cancer metastasis and migration of breast cancer cells [63].
*FIP1L1*	1	HPEpiC	−	The fusion of the gene encoding the Pre-mRNA 3'-end-processing factor FIP, with the *PDGFRA* gene or the *RARA* gene generate a novel tyrosine kinase due to a interstitial chromosomal deletion [64]. These associations have been linked to the pathogenesis of leukemias [65].
*HSPA5*	1	HOSEpiC	Tumour progression	78 kDa glucose-regulated protein (GRP-78) or Heat shock 70 kDa protein 5 has been proposed as a potential biomarker for predicting high-risk endometrial carcinoma [66], as well as positive regulator of cellular migration [67].
*KRT7*	19	HPEpiC	Cytoskeleton	Keratin, type II cytoskeletal 7. The keratin *KRT7* gene has been found to be hyper-methylated in prostate cancer [33].
*NEXN*	11	HOSEpiC	Cytoskeleton Cell motility	Nexilin is an F-actin associated protein that stimulates cell motility (Wang *et al.*, 2005) and NEXN is down regulated after radiation of prostate adenocarcinoma cells [68].
*PCOLCE*	1	HOSEpiC	Tumour progression	Procollagen C-endopeptidase enhancer 1 (PCPE-1) binds procollagen, potentiating its cleavage by specific proteinases, and might be involved in tumour growth [69] .
*RPL29*	2	HOSEpiC	Tumour progression	60S ribosomal protein L29 has been related to tumoral events as angiogenesis and cell proliferation [70].
*RPS20*	1	HOSEpiC	Cellular signalling	40S ribosomal protein S20 binds the RING-type E3 ubiquitin ligases Mdm2 and MdmX therefore regulating the Mdm2-p53-MdmX network [71].
*SPINT1*	2	HPEpiC	Cellular signalling	Kunitz-type protease inhibitor 1 (or hepatocyte growth factor activator inhibitor type 1, HAI-1) malfunction promotes intestinal carcinogenesis by activating NF-κB signalling [72]. Low HAI-1 expression is a significant predictor for poor prognosis in prostate cancer [73].
*UBE2I*	1	HPEpiC	Tumour progression	SUMO-conjugating enzyme UBC9, plays a major role in sumoylation [74]. UBE2I expression is increased in several cancers as in epithelial ovarian cancer (EOC) [75], colon and prostate neoplasias and adenocarcinomas [76]. Moreover, overexpression of UBE2I has also been related to migration, invasion and proliferation in a lung cancer cell line [77] and in ovarian cancer [78]
*ZNF428*	11	HOSEpiC	Tumour progression	Zinc finger protein 428 interacts with Bcl2-associated athanogene 3 (BAG3) protein, which is related to cancerous processes. BAG3 can modulate apoptosis, autophagy, mechanotransduction, cytoskeleton organization, and motility [79]. Moreover, BAG3 regulates epithelial-mesenchymal transition (EMT) and angiogenesis and its knockdown reduced migration and invasion in cancerous cells [80].

*N*: repetitive identification during Y2H screening

Interestingly, all the identified HMGB1 binding proteins have been reported to be involved in cellular proliferation and cancer progression, as detailed in Table 1, some of them specifically in ovary or prostate cancer. This is the case for keratin KRT7 [33] and SPINT1, a membrane-bound serine protease inhibitor expressed on the epithelial cell surface [34–36]. Keratins form obligate heteropolymers between one type I and one type II keratin; the keratin prey found in our analyses is from type II and their human *in vivo* partners from type I is KRT18, which has been proposed as biomarker in prostate carcinoma [37]. Since keratin expression is tissue-specific, they are extensively used as diagnostic tumor markers. Moreover, they are also active players in cancer progression and metastasis, as well as multifunctional regulators of epithelial tumorigenesis [37]. In prostate cancer, SPINT1 regulates matriptase, a membrane-associated serine protease that activates prostatin by regulation of HAI-1, which is an endogenous inhibitor [36]. A recent study has demonstrated that ovarian cancer cell metastasis and invasion are dependent on upregulation of matriptase [34].

### HMGB2 binding proteins in HPEpiC and HOSEpiC cells

The proteins interacting with HMGB2 as detected by Y2H (Table 2) can be classified in several groups: a) apoptosis-related proteins: ZNF622, EIF1, SAMM50 and ZNF428; b) cell motility: C1QBP, MIEN1 and NEXN; c) cellular proliferation: NAP1L1, CACTIN, ARCN1, ZNF668, NCBP3, H3F3A, HDLBP, HACD3 and FEZ1. None of these interactors has previously been reported.

**Table 2:** HMGB2 preys in HOSEpic and HPEpiC cells

Gene	*N*	Cells	Function	Relation to cancer
*ARCN1*	1	HPEpiC	Cell proliferation	*ARCN1* encodes the Coatomer subunit delta and its depletion inhibits growth of cancer cell lines without affecting normal cell growth and survival [81]. It is functionally related to the retrograde transport mediating intracellular protein traffic [82]. It is targeted by miR-361-5p [83].
*C1QBP*	12	HPEpiC	Cell motility	Previously described in Table 1.
*CACTIN*	1	HPEpiC	Cell proliferation	Cactin forms part of a family of conserved eukaryotic proteins, which are required for multiple processes including cell proliferation and genome stability maintenance by allowing faithful splicing of specific pre-mRNAs [84] and it has been reported to inhibit NFκB- and TLR-mediated transcription [85].
*EIF1/SUI1*	1	HPEpiC	Apoptosis	Previously described in Table 1.
*H3F3A*	1	HPEpiC	Cell proliferation	Mutations in the gene codifying Histone H3.3, have been associated to glioma [86]. Moreover, overexpression of this histone variant has been associated with lung cancer progression by activating metastasis-related genes [87].
*HACD3*	1	HOSEpiC	Cell proliferation	Very-long-chain (3R)-3-hydroxyacyl-CoA dehydratase 3, HACD3 plays a role in Rac1 signalling pathways [88] a small GTPase widely related to tumoral processes [89].
*HDLBP*	1	HOSEpiC	Cell proliferation	The high density lipoprotein-binding protein, Vigilin, may be involved in cell proliferation [90] and in chromosomal condensation and segregation [91]. HDLBP is upregulated in hepatocellular carcinomas [92].
*LZTS1/ FEZ1*	4	HOSEpiC	Cell proliferation	Leucine zipper putative tumor suppressor 1. The LZTS1 tumor suppressor gene 1 (*FEZ1*) inhibits cell proliferation and tumor growth in colorectal cancer cells, in part via suppression of AMT-mTOR [93].
*MIEN1*	1	HPEpiC	Cell motility	Migration and invasion enhancer 1 is involved in filopodia formation and tumor cell migration. Differently expressed in normal and cancer cells [94]. Related to prostate cancer [38].
*NAP1L1*	1	HPEpiC	Cell proliferation	Nucleosome assembly protein 1-like is a chaperone for histone exchange in nucleosomes and it promotes cell proliferation [95].
*NCBP3*	1	HOSEpiC	Cell Proliferation	Nuclear cap-binding protein subunit 3 associates with components of the mRNA processing machinery and contributes to poly (A) RNA export. With Nuclear cap-binding protein subunit 1 forms an alternative cap-binding complex in higher eukaryotes [96]. Silencing of NCBP1 causes deregulated expression of several hundred genes and a reduction in the cell proliferation rate [97].
*NEXN*	4	HOSEpiC	Cell motility Cytoskeleton	Previously described in Table 1.
*SAMM50*	2	HOSEpiC	Apoptosis	Sorting and assembly machinery component 50 homolog participates in mitochondrial respiratory chain complexes assembly and mitochondrial morphology. In apoptotic cells, mitochondrial fission requires the cytoplasmic dynamin-related protein, DRP1, a GTPase protein responsible for mitochondrial division that translocates to the nucleus [98]. DRP1 is involved in apoptosis in prostate cancer cells [42].
*ZNF428*	15 1	HOSEpiC HPEpiC	Apoptosis	Previously described in Table 1
*ZNF622*	1	HPEpiC	Apoptosis	Zinc finger protein 622 is involved in apoptosis and regulation of JNK cascade. Interacts with Apoptosis signal-regulating kinase 1(ASK1); ASK family members are activated by a wide variety of stressors, and they regulate various cellular responses, such as cell proliferation, inflammation and apoptosis in cancer development [99].
*ZNF668*	4	HPEpiC	Cell proliferation	Zinc finger protein 668 suppresses breast cancer cell proliferation. *ZNF668* is a breast tumor suppressor gene. Zinc finger protein 668 physically interacts with and regulates p53 stability [100]. It promotes H2AX acetylation after DNA damage and is a regulator of DNA repair [35].

*N*: repetitive identification during Y2H screening.

All these HMGB2 binding proteins have also been reported to be involved in cellular proliferation and cancer progression as detailed in Table 2, and in some cases specifically associated to ovary or prostate cancer. MIEN1 (c17crf37) maintains the plasticity of the dynamic membrane-associated actin cytoskeleton, and its overexpression in prostate cancer functionally enhances migration and invasion of tumor cells [38]. NAP1L1 (nucleosome-assembly-protein 1), a chaperone for histone exchange in nucleosomes, is the only ATP-dependent chromatin remodeler essential for transcription-coupled nucleotide excision DNA repair [39] and it is a candidate biomarker identified in primary ovarian tumors that respond to cytotoxic gold compounds [40]. SAMM50 has been linked to apoptosis through its role in the targeting of the mitochondrial channel VDAC [41]. In addition, SAMM50 interacts with DRP1, a dynamin-like protein involved in apoptosis in prostate cancer cells [42]. FEZ1 is a mitotic regulator implicated in cancer [43] that is associated with microtubule components [44]. It is a leucine-zipper protein, and its expression is undetectable in 60% of ephitelial human tumors, including prostate [45]. It has been suggested as a tumor suppressor gene in prostate and other cancers [46] and therefore its loss may contribute to tumor progression, as has been also found in ovarian carcinogenesis [47].

### Identification of HMGB1 binding proteins by Y2H screening of a healthy ovarian tissue library

Healthy ovarian tissue obtained after biopsy was used for RNA extraction and construction of cDNA libraries. The library was screened for interactions using HMGB1 as bait, as described in the materials and methods section. The efficiency obtained in the preparation of the tissue-based libraries was lower too. As observed with HPEpiC and HOSEpiC libraries, HMGB1 binding proteins identified from healthy ovarian tissue libraries are also all involved in cellular proliferation and cancer progression, as detailed in Table 3. Four of them have been reported to be specifically related to ovary or prostate cancer, namely CCAR1 [48] KRT7 [49] already identified with the other libraries, MT2A, a metallothionein that promotes the survival of human prostate cancer cells [50] and C3, Complement C3 protein, that contributes to ovarian tumoral cell proliferation, migration and invasion [51].

**Table 3:** HMGB1 Y2H preys in a library from ovarian tissue

Gene	*N*	Function	Relation to cancer
*C3*	1	Cell Proliferation	Secretion of Complement C3 protein in ovarian cancer cells promotes cell proliferation, invasion, and migration [51].
*CCAR1*	3	Apoptosis	Cell division cycle and apoptosis regulator 1 regulates cell growth and apoptosis by serving as a co-activator of several genes including steroid nuclear receptors and tumor suppressor p53 [48].
*KRT7*	5	Cytoskeleton	KRT7 7, KRT18 and KRT19 are differentially expressed in circulating tumor cells in ovarian cancer patients [49].
*MT2A*	1	Apoptosis	MT2A is upregulated in chemotherapy resistant ovarian tumors, and also in human prostate cell lines [50].
*RPS12*	1	Cell Proliferation	RPS12 encodes the 40S ribosomal protein S12. RPS12 expression changes in breast cancer [101].

*N*: repetitive identification during Y2H screening.

### Cross validation of HMGB1 and HMGB2 interactions identified by different strategies

In this study we used two complementary techniques and three epithelial cell lines to investigate HMGB1 and HMGB2 partners in the context of epithelial tissue. MS-based strategies are well-recognized methods to investigate interacting proteins or protein complexes [52]. However, this technique has several limitations, and the interactions detected could be direct or mediated by other interacting proteins. In addition, expression of the proteins involved in the interaction might be dependent of cell signaling pathways, which are not known *a priori* and therefore cannot be accounted for in the experimental design. By contrast, the Y2H technology allows the co-expression of the two partners of the interaction, independently of cellular signals, bypassing this limitation, but has other drawbacks, like heterologous expression in yeast, or conditioned nuclear localization of bait and prey due to their fusion to a transcriptional factor. Interactions identified in this study that are cross-validated because they were detected either using both Co-IP/MS and Y2H approaches, or detected at least in two different epithelial cells include CCAR1, KRT7, RPS12, RPS20, and ZNF428. In addition, the Identification of PARP1 by mass spectrometry after IP with HMGB1 antibody was validated by western blot (Figure 1).

The data obtained in our study reveal that, in epithelial cells, HMGB1 interacts with many nuclear proteins but also with proteins that form part of the cytoskeleton, cell-adhesion structures and others related to intracellular protein translocation, a process which is involved in diverse cellular functions including cellular migration, secretion, apoptosis and cell survival. Among the cross-validated HMGB1 interactions are nuclear proteins affecting gene expression, such as Cell division cycle and apoptosis regulator 1 (CCAR1) [53]. CCAR1 is a coactivator necessary for recruitment of the Mediator complex by nuclear receptors and it regulates expression of key proliferation inducing genes. CCAR1 also functions as a p53 coactivator, which explains its broader role in transcriptional regulation. Extra-nuclear proteins were also found, like Cytokeratin 7, one of the intermediate filament proteins that constitute the cytoskeleton of numerous types of epithelial cells [54]. CCAR1 and KRT7 were found to interact with HMGB1 in two different cell types, CCAR1 in HOSEpiC and healthy ovarian tissue cells, and KRT7 in HPEpiC and healthy ovarian tissue cells. Only one protein interacting with HMGB2 was cross-validated in our study, the Zinc Finger Factor 428 (ZNF428), an uncharacterized zinc finger protein detected by using HPEpiC and HOSEpiC cells in the Y2H strategy.

Since HGMB1 and HMGB2 have sequences with 82.3 % similarity, we expected that the two baits would have common targets, but we found only four, ZNF428, EIF1, C1QBP and Nelin (NEXN), an F-actin associated protein involved in cell motility [55]. Intersection of known interactions for HMGB1 (89 hits) and HMGB2 (55 hits) in BioGRID reveals only 19 overlapping proteins, which suggests that the specificity of these proteins for their respective partners is high.

The proteins interacting with HMGB1 or HMGB2 in prostate and ovarian epithelial cells identified in our study are involved in cellular processes which are altered in cancerous cells, and hence provide a useful resource for further investigation of the complex roles of HMGB1/2 in cancer [56]. Our data should also be of interest in future clinical trials to identify potential biomarkers of cancer diagnosis and progression, as well as pharmacological targets for the development of new therapy approaches for cancers of epithelial origin.

## MATERIALS AND METHODS

### Yeast strains

*Sacchacomyces cerevisiae* strains used in this study were: Y187 (MATα, *ura3-52, his3-200, ade2-101, trp1-901, leu2-3, 112, gal4Δ, gal80Δ, met-, URA3::GALUAS-GAL1TATA-LacZ MEL1)* and Y2HGold (MATa, *trp1-901, leu2-3, 112, ura3-52, his3-200, gal4Δ, gal80Δ, LYS2:: GAL1UAS-Gal1TATA-His3, GAL2UAS-Gal2TATA-Ade2 URA3:: MEL1UAS-Mel1TATA, AUR1-C MEL1)*

### Library construction

cDNA libraries were constructed using Make Your Own “Mate&Plate” Library System (Clontech, CA Laboratories Inc., CA, USA). 1.0–2.0 µg of total RNA from HOSEpiC, and HPEpiC provided by Innoprot (Spain) and from healthy ovarian tissue provided by Biobanco de Andalucía (Spain) was used to construct each library. cDNA was generated using Clontech SMART technology following the manufacturer´s instructions. A degenerated oligo-dT primer: CDS III Primer (5′-ATTCTAGAGGCCGAGGCGGCCGACATG-d(T)30VN-3′, where *N* = A, G, C or T and V = A, G or C) was used for first strand cDNA synthesis, after reverse transcription SMART III-modified Oligo (5′-AAGCAGTGGTATCAACGCAGAGTGGCCATTATGGCCGGG-3′) was incorporated to the cDNA. Double strand cDNA was amplified using Long Distance PCR (LD-PCR) after which ds cDNA molecules over 200 pb were purified with CHROMA SPINTM+ TE-400 Columns.

To prepare each library, competent Y187 yeast cells were cotransformed with 20 µl of the ds cDNA (2–5 µg) and 3µg of pGADT7-Rec (*Sma*I-linearalized) following the Yeastmaker Yeast Transformation System 2 (Clontech Laboratories Inc., CA, USA) protocol. pGADT7-Rec vector is suitable for expressing a protein fused to the GAL4 activation domain (AD) under the constitutive *ADH1* promoter (PADH1). This vector contains *LEU2* nutritional marker for selection in yeast (www.clontech.com/xxclt_ibcGetAttachment.jsp?cItemId=17643). Cells were plated on CM/-Leu plates for selection and incubated at 30° C for 3–4 days. All the colonies were harvested with freezing medium (YPDA/25% glycerol) and pooled together. The library was divided in 1 ml aliquots and stored at −80° C.

### Bait construction

The bait clones were generated fusing the *HMGB1* or *HMGB2* genes to the GAL4 DNA binding domain (DNA-BD) of the plasmid pGBKT7 (Clontech Laboratories Inc., CA, USA). This vector allows high level expression of fusion proteins due to the constitutive *ADH1* promoter (PADH1) and carries the *TRP1* gene that is used for auxotrofic selection in yeast (www.clontech.com/xxclt_ibcGetAttachment.jsp?cItemId=17639). *HMGB1* and *HMGB2* were amplified from commercial vectors containing the *HMGB1* and *HMGB2* complete ORFs (OriGene, USA). Primers used to amplify the bait genes were designed with the following sequences: 5′-CATGGAGGCCGAATTCATGGGCAAAGGAGATCCTAAGAAG-3′ and 5′-GCAGGTCGACGGATCCTTATTCATCATCATCATCTTCTTCTTCATC-3′ for *HMGB1* and 5′-CATGGAGGCCCCGGGATGGGTAAAGGAGACCCCAACAAG-3′ and 5′-GCAGGTCGACGGATCCTTATTCTTCATCTTCATCCTCTTC-3′ for *HMGB2*. *HMGB1* was cloned in the PGBKT7-BD plasmid using the In-Fusion Cloning Kit (Clontech Laboratories Inc, CA, USA). *HMGB2* construct was obtained by digestion of both the PGBKT7-BD and the *HMGB2* PCR product with *Sma*I and *Bam*HI and subsequent ligation. The constructs were sequenced to confirm correct insertion of each gene. Y2HGold cells were transformed with pGBKT7-BD bait plasmids and plated in CM/-Trp to select for selection of bait plasmids. Prior to the two-hybrid assays, both baits were tested for autoactivation to confirm that neither HMGB1 nor HMGB2 autonomously activate the reporter genes in Y2HGold.

### Yeast two-hybrid library screening

HMGB1 and HMGB2 interacting partners were assessed using Matchmaker Gold Yeast Two-Hybrid System (Clontech Laboratories Inc., CA, USA). For each Y2H screening 1 ml of the library was combined with the bait strain following the manufacturer´s instructions. In short, the mating between both strains was performed in 2×YPDA medium during 24 h at 30° C with slow shaking (30–50 rpm). The efficiencies in the constructions of libraries were in the range recommended in the kit, although minor in ovarian tissue libraries than in HOSEpiC or HPEpiC cell libraries. After the mating, cells were plated in CM/-Trp/-Leu/-His. In each two hybrid assay more than 1 million clones (diploids) were screened, calculated as indicated by the kit. The clones growing in CM/-Trp/-Leu/-His were tested in two more media: CM/-Trp/-Leu/-Ade and CM/-Trp/-Leu/X-α-Gal. Clones that could grow in all specific depleted media and displayed α-galactosidase activity contained the cDNA of a possible interacting partner in the pGADT7-Rec vector. In order to identify the encoded proteins the prey plasmid was rescued and amplified in bacteria, and the inserted cDNA was sequenced using the T7 sequencing primer (5′-TAATACGACTCACTATAGGG-3′). The sequences were analyzed using Blastn from the NCBI (https://blast.ncbi.nlm.nih.gov/Blast.cgi?PROGRAM=blastn&PAGE_TYPE=BlastSearch&LINK_LOC=blasthome).

### Cell culture

Immortalized human benign prostate epithelial cells PNT2 (kindly provided by Dr. Inés Díaz-Laviada Mauret) were grown in RPMI-1640 (Thermo Fisher Scientific, Inc., MA, USA), supplemented with 10% fetal bovine serum (Thermo Fisher Scientific, Inc.) and 1% penicillin-streptomycin (Thermo Fisher Scientific, Inc.). All cells were maintained in standard cell culture conditions (37° C and 5% CO2 in a humidified incubator). Cells were regularly tested for mycoplasma.

### Immunoprecipitation

Total protein from PNT2 cells was extracted in lysis buffer (50 mM Tri-HCl pH 8.0, 150 mM NaCl, 0.1% NP-40, 1 mM EDTA, 2 mM MgCl2 and Complete^™^ Mini, EDTA-free Protease Inhibitor Cocktail (Roche , Basel, Swietzerland) and incubated for 30 min at 4° C with Benzonase^®^ Nuclease (Sigma-Aldrich MO, USA) to eliminate nucleic acids from the lysates. Total protein was quantified using the Bradford reagent. Prior to the immunoprecipitation, Protein G-Dynabeads (Invitrogen, MA, USA) were crosslinked to 40 µg of HMGB1 antibody (ab18256, Abcam, Cambridge, UK) or anti-rabbit IgG antibody (Millipore, Darmstadt, Germany) as previously described. For each immunoprecipitation 2.5–3 mg of protein were incubated for 4 h at 4° C with the corresponding HMGB1 or control rabbit antibody-linked beads. Nonspecific binding proteins were removed by four washes with IPP150 buffer (10 mM Tris-HCl pH8.0, 150 mM NaCl and 0.1% NP-40) and four washes with 50 mM ammonium bicarbonate. On-bead digestion was carried out overnight at 37° C with trypsin (sequencing grade, Roche Basel, Swietzerland). Peptides were then collected, acidified with formic acid, filtered through Millipore Multiscreen HTS plates and dried in a Speed Vac (Thermo Fisher Scientific, MA, USA). Peptides were then resuspended in 20 mM TCEP and formic acid was added to a final concentration of 0.5%.

### Mass spectrometry and data analysis

Peptides were analysed with online nanoLC-MS/MS on an Orbitrap Velos mass spectrometer coupled with an Ultimate 3000 RSLCnano System. Samples were first loaded and desalted on a nanotrap (100 µm id × 2 cm) (PepMap C18, 5 µ) at 10 µL/min with 0.1% formic acid for 10 min and then separated on an analytical column (75 µm id × 25 cm) (PepMap C18, 2 µ) over a 90 min linear gradient of 5–42% B (where B = 80% CH3CN/0.1% formic acid) at 300 nL/min, and the total cycle time was 120 min (for AB01 to AB06), or a 120 min linear gradient of 4–32% CH3CN/0.1% formic acid at 300 nL/min, and the total cycle time was 150 min (from AB07). The Orbitrap Velos was operated in standard data-dependent acquisition. The survey scans (m/z 380–1500) were acquired in the Orbitrap at a resolution of 30,000 at m/z 400, and one microscan was acquired per spectrum. The 10 most abundant multiply charged ions with a minimal intensity of 2000 counts were subject to MS/MS in the linear ion trap at an isolation width of 2 Th. Dynamic exclusion width was set at ± 10 ppm for 45 s. The automatic gain control target value was regulated at 1 × 10^6^ for the Orbitrap and 5000 for the ion trap, with maximum injection time at 200 ms for Orbitrap and 100 ms for the ion trap, respectively.

The raw files were processed with Proteome Discoverer v1.4 (Thermo Fisher Scientific, MA, USA). Database searches were performed with Mascot (Matrix Science) against the human Uniprot database (2014, 77606 entries) and an in-house contaminant database. The search parameters were: trypsin/P digestion, 2 missed cleavages, 10 ppm mass tolerance for MS, 0.5 Da mass tolerance for MS/MS, with variable modifications of acetyl (N-terminal), carbamidomethyl (C), N-formylation (protein), oxidation (M), and pyro-glu (N-term Q). Database search results were refined through processing with Percolator (significance threshold < 0.05, FDR < 1%). Protein identification required at least one high-confidence peptide (FDR < 1%) with a minimum score of 20. External contaminants (albumin, casein, trypsin,) were removed from protein lists before further analysis. Keratins were not removed, as they could potentially represent true interactors.

To discriminate specific from unspecific interactions, the identified proteins in each immunoprecipitation (HMGB1 and IgG antibody) were analyzed with the Significance Analysis of INTeractome (SAINT) score SAINTexpress [17, 18]. SAINT models the distribution of true and false interactions using label-free quantitative data from affinity purification coupled to mass spectrometry bait and negative control experiments, and calculates the posterior probability of a true interaction for each prey-bait pair from the quantitative data. Results from each experiment were analyzed using their corresponding negative control. Preys with SAINT probability score cut-off of 1 detected by at least two unique peptides were deemed high confidence HMGB1 interacting proteins and further analyzed for functional significance. The mass spectrometry proteomics data have been deposited to the ProteomeXchange Consortium via the PRIDE [57] partner repository with the dataset identifier PXD007867.

### Western blot analysis

Immunoprecipitations previous to western blot were carried out as above described but at minor scale, starting with 1 mg of total protein and 4 µg of antibody. Proteins were eluted by incubation in 1 × LDS loading buffer (Thermo Fisher Scientific, MA, USA), containing 350 mM β-mercaptoethanol, at 95° C for 10 min. Protein samples were run on 10% SDS-PAGE gels at 80 V for 20 minutes and at 200 V for the next 45–60 minutes, and then transferred onto a PVDF membrane at 0.2 A for 1 hour. Membranes were blocked by incubating with 5% nonfat dry milk for 1 hour at room temperature (RT) and then incubated with primary antibodies: anti-HMGB1 (anti-HMGB1 antibody ChIP grade ab18256 from Abcam Cambridge, UK) or anti-PARP1 (ab6079​ from Abcam Cambridge, UK) in PBST (containing 0.1% Tween 20) overnight at 4° C. After incubation with the secondary antibody, anti-protein G - HRP conjugate 1/5000 (Millipore, Darmstadt, Germany), protein bands were detected using LuminataTMCrescendo Western HRP Substrate (Millipore Darmstadt, Germany) according to manufacturer’s instructions. Bio-Rad (CA, USA) ChemiDocTM imager was used for chemiluminescence detection.

## SUPPLEMENTARY MATERIALS





## Abbreviations

CoI: Co-Immunoprecipitation
CM: Complete Medium
ER: Endoplasmic Reticulum
FDR: False Discovery Rate
FEDER: Fondo Europero para el Desarrollo Regional
GO: Gene Ontology
HMGB: High Mobility Group B
HOSEpiC: Human Ovarian Surface Epithelial Cells
HPEpiC: Human Prostate Epithelial Cells
INIBIC: Instituto de Investigaciones Biomédicas de A Coruña
MS: mass spectrometry
oligo: oligonucleotide
ORF: Open Reading Frame
SAINT: Significance Analysis of INTeractome
Y2H: Yeast two Hybrid assays

## References

[1] Berman JJ (2004). Tumor taxonomy for the developmental lineage classification of neoplasms. BMC Cancer.

[2] Lange SS, Vasquez KM (2009). HMGB1: the jack-of-all-trades protein is a master DNA repair mechanic. Molecular Carcinogenesis.

[3] Tang D, Kang R, Zeh HJ, Lotze MT (2010). High-mobility group box 1 and cancer. Biochimica et Biophysica Acta.

[4] Gnanasekar M, Thirugnanam S, Ramaswamy K (2009). Short hairpin RNA (shRNA) constructs targeting high mobility group box-1 (HMGB1) expression leads to inhibition of prostate cancer cell survival and apoptosis. International Journal of Oncology.

[5] Chen J, Liu X, Zhang J, Zhao Y (2012). Targeting HMGB1 inhibits ovarian cancer growth and metastasis by lentivirus-mediated RNA interference. Journal of Cellular Physiology.

[6] Chen J, Xi B, Zhao Y, Yu Y, Zhang J, Wang C (2012). High-mobility group protein B1 (HMGB1) is a novel biomarker for human ovarian cancer. Gynecologic Oncology.

[7] Zhao CB, Bao JM, Lu YJ, Zhao T, Zhou XH, Zheng DY, Zhao SC (2014). Co-expression of RAGE and HMGB1 is associated with cancer progression and poor patient outcome of prostate cancer. American Journal of Cancer Research.

[8] Gatla HR, Singha B, Persaud V, Vancurova I (2014). Evaluating cytoplasmic and nuclear levels of inflammatory cytokines in cancer cells by western blotting. Methods Mol Biol.

[9] Jung Y, Lippard SJ (2003). Nature of full-length HMGB1 binding to cisplatin-modified DNA. Biochemistry.

[10] Ohndorf UM, Rould MA, He Q, Pabo CO, Lippard SJ (1999). Basis for recognition of cisplatin-modified DNA by high-mobility-group proteins. Nature.

[11] Lange SS, Reddy MC, Vasquez KM (2009). Human HMGB1 directly facilitates interactions between nucleotide excision repair proteins on triplex-directed psoralen interstrand crosslinks. DNA Repair.

[12] Huang JC, Zamble DB, Reardon JT, Lippard SJ, Sancar A (1994). HMG-domain proteins specifically inhibit the repair of the major DNA adduct of the anticancer drug cisplatin by human excision nuclease. Proceedings of the National Academy of Sciences of the United States of America.

[13] Yusein-Myashkova S, Stoykov I, Gospodinov A, Ugrinova I, Pasheva E (2016). The repair capacity of lung cancer cell lines A549 and H1299 depends on HMGB1 expression level and the p53 status. Journal of Biochemistry.

[14] Karsch-Bluman A, Amoyav B, Friedman N, Shoval H, Schwob O, Ella E, Wald O, Benny O (2017). High mobility group box 1 antagonist limits metastatic seeding in the lungs via reduction of cell-cell adhesion. Oncotarget.

[15] Liu PL, Liu WL, Chang JM, Chen YH, Liu YP, Kuo HF, Hsieh CC, Ding YS, Chen WW, Chong IW (2017). MicroRNA-200c inhibits epithelial-mesenchymal transition, invasion, and migration of lung cancer by targeting HMGB1. PloS One.

[16] Zhang R, Li Y, Wang Z, Chen L, Dong X, Nie X (2015). Interference with HMGB1 increases the sensitivity to chemotherapy drugs by inhibiting HMGB1-mediated cell autophagy and inducing cell apoptosis. Tumour biology.

[17] Choi H, Liu G, Mellacheruvu D, Tyers M, Gingras AC, Nesvizhskii AI (2012). Analyzing protein-protein interactions from affinity purification-mass spectrometry data with SAINT. Current Protocols in Bioinformatics.

[18] Teo G, Liu G, Zhang J, Nesvizhskii AI, Gingras AC, Choi H (2014). SAINTexpress: improvements and additional features in Significance Analysis of INTeractome software. Journal of Proteomics.

[19] Stark C, Breitkreutz BJ, Reguly T, Boucher L, Breitkreutz A, Tyers M (2006). BioGRID: a general repository for interaction datasets. Nucleic Acids Research.

[20] Anggayasti WL, Mancera RL, Bottomley S, Helmerhorst E (2016). The effect of physicochemical factors on the self-association of HMGB1: A surface plasmon resonance study. Biochimica et Biophysica Acta.

[21] Szklarczyk D, Morris JH, Cook H, Kuhn M, Wyder S, Simonovic M, Santos A, Doncheva NT, Roth A, Bork P, Jensen LJ, von Mering C (2017). The STRING database in 2017: quality-controlled protein-protein association networks, made broadly accessible. Nucleic Acids Research.

[22] Stros M (2010). HMGB proteins: interactions with DNA and chromatin. Biochimica et Biophysica Acta.

[23] Thomas JO, Stott K (2012). H1 and HMGB1: modulators of chromatin structure. Biochemical Society Transactions.

[24] Das D, Scovell WM (2001). The binding interaction of HMG-1 with the TATA-binding protein/TATA complex. The Journal of Biological Chemistry.

[25] Thomas MC, Chiang CM (2006). The general transcription machinery and general cofactors. Critical Reviews in Biochemistry and Molecular Biology.

[26] Banerjee S, Kundu TK (2003). The acidic C-terminal domain and A-box of HMGB-1 regulates p53-mediated transcription. Nucleic Acids Research.

[27] Kang R, Tang D, Schapiro NE, Loux T, Livesey KM, Billiar TR, Wang H, Van Houten B, Lotze MT, Zeh HJ (2014). The HMGB1/RAGE inflammatory pathway promotes pancreatic tumor growth by regulating mitochondrial bioenergetics. Oncogene.

[28] Lv G, Wu M, Wang M, Jiang X, Du J, Zhang K, Li D, Ma N, Peng Y, Wang L, Zhou L, Zhao W, Jiao Y (2017). miR-320a regulates high mobility group box 1 expression and inhibits invasion and metastasis in hepatocellular carcinoma. Liver international.

[29] Li ML, Wang XF, Tan ZJ, Dong P, Gu J, Lu JH, Wu XS, Zhang L, Ding QC, Wu WG, Rao LH, Mu JS, Yang JH (2012). Ethyl pyruvate administration suppresses growth and invasion of gallbladder cancer cells via downregulation of HMGB1-RAGE axis. International Journal of Immunopathology and Pharmacology.

[30] Pellegrini L, Xue J, Larson D, Pastorino S, Jube S, Forest KH, Saad-Jube ZS, Napolitano A, Pagano I, Negi VS, Bianchi ME, Morris P, Pass HI (2017). HMGB1 targeting by ethyl pyruvate suppresses malignant phenotype of human mesothelioma. Oncotarget.

[31] Lázaro-Ibáñez E, Lunavat TR, Jang SC, Escobedo-Lucea C, Oliver-De La Cruz J, Siljander P, Lötvall J, Yliperttula M (2017). Distinct prostate cancer-related mRNA cargo in extracellular vesicle subsets from prostate cell lines. BMC Cancer.

[32] Sheller-Miller S, Urrabaz-Garza R, Saade G, Menon R (2017). Damage-Associated molecular pattern markers HMGB1 and cell-free fetal telomere fragments in oxidative-Stressed amnion epithelial cell-derived exosomes. Journal of Reproductive Immunology.

[33] Ibragimova I, Ibanez de Caceres I, Hoffman AM, Potapova A, Dulaimi E, Al-Saleem T, Hudes GR, Ochs MF, Cairns P (2010). Global reactivation of epigenetically silenced genes in prostate cancer. Cancer Prevention Research.

[34] Sun P, Jiang Z, Chen X, Xue L, Mao X, Ruan G, Song Y, Mustea A (2016). Decreasing the ratio of matriptase/HAI1 by downregulation of matriptase as a potential adjuvant therapy in ovarian cancer. Molecular Medicine Reports.

[35] Hu R, Wang E, Peng G, Dai H, Lin SY (2013). Zinc finger protein 668 interacts with Tip60 to promote H2AX acetylation after DNA damage. Cell Cycle.

[36] Warren M, Twohig M, Pier T, Eickhoff J, Lin CY, Jarrard D, Huang W (2009). Protein expression of matriptase and its cognate inhibitor HAI-1 in human prostate cancer: a tissue microarray and automated quantitative analysis. Applied Immunohistochemistry and Molecular Morphology.

[37] Karantza V (2011). Keratins in health and cancer: more than mere epithelial cell markers. Oncogene.

[38] Dasgupta S, Wasson LM, Rauniyar N, Prokai L, Borejdo J, Vishwanatha JK (2009). Novel gene C17orf37 in 17q12 amplicon promotes migration and invasion of prostate cancer cells. Oncogene.

[39] Lee JY, Lake RJ, Kirk J, Bohr VA, Fan HY, Hohng S (2017). NAP1L1 accelerates activation and decreases pausing to enhance nucleosome remodeling by CSB. Nucleic Acids Research.

[40] Guidi F, Puglia M, Gabbiani C, Landini I, Gamberi T, Fregona D, Cinellu MA, Nobili S, Mini E, Bini L, Modesti PA, Modesti A, Messori L (2012). 2D-DIGE analysis of ovarian cancer cell responses to cytotoxic gold compounds. Molecular bioSystems.

[41] Kozjak-Pavlovic V, Ross K, Benlasfer N, Kimmig S, Karlas A, Rudel T (2007). Conserved roles of Sam50 and metaxins in VDAC biogenesis. EMBO Reports.

[42] Kaddour-Djebbar I, Choudhary V, Brooks C, Ghazaly T, Lakshmikanthan V, Dong Z, Kumar MV (2010). Specific mitochondrial calcium overload induces mitochondrial fission in prostate cancer cells. International Journal of Oncology.

[43] Vecchione A, Croce CM, Baldassarre G (2007). Fez1/Lzts1 a new mitotic regulator implicated in cancer development. Cell Division.

[44] Ishii H, Vecchione A, Murakumo Y, Baldassarre G, Numata S, Trapasso F, Alder H, Baffa R, Croce CM (2001). FEZ1/LZTS1 gene at 8p22 suppresses cancer cell growth and regulates mitosis. Proceedings of the National Academy of Sciences of the United States of America.

[45] Ishii H, Baffa R, Numata SI, Murakumo Y, Rattan S, Inoue H, Mori M, Fidanza V, Alder H, Croce CM (1999). The FEZ1 gene at chromosome 8p22 encodes a leucine-zipper protein, and its expression is altered in multiple human tumors. Proceedings of the National Academy of Sciences of the United States of America.

[46] Cabeza-Arvelaiz Y, Sepulveda JL, Lebovitz RM, Thompson TC, Chinault AC (2001). Functional identification of LZTS1 as a candidate prostate tumor suppressor gene on human chromosome 8p22. Oncogene.

[47] Califano D, Pignata S, Pisano C, Greggi S, Laurelli G, Losito NS, Ottaiano A, Gallipoli A, Pasquinelli R, De Simone V, Cirombella R, Fusco A, Chiappetta G (2010). FEZ1/LZTS1 protein expression in ovarian cancer. Journal of Cellular Physiology.

[48] Muthu M, Cheriyan VT, Rishi AK (2015). CARP-1/CCAR1: a biphasic regulator of cancer cell growth and apoptosis. Oncotarget.

[49] Kolostova K, Pinkas M, Jakabova A, Pospisilova E, Svobodova P, Spicka J, Cegan M, Matkowski R, Bobek V (2016). Molecular characterization of circulating tumor cells in ovarian cancer. American Journal of Cancer Research.

[50] Yamasaki M, Nomura T, Sato F, Mimata H (2007). Metallothionein is up-regulated under hypoxia and promotes the survival of human prostate cancer cells. Oncology Reports.

[51] Cho MS, Vasquez HG, Rupaimoole R, Pradeep S, Wu S, Zand B, Han HD, Rodriguez-Aguayo C, Bottsford-Miller J, Huang J, Miyake T, Choi HJ, Dalton HJ (2014). Autocrine effects of tumor-derived complement. Cell Reports.

[52] Gingras AC, Wong CJ (2016). Proteomics approaches to decipher new signaling pathways. Current Opinion in Structural Biology.

[53] Kim JH, Yang CK, Heo K, Roeder RG, An W, Stallcup MR (2008). CCAR1, a key regulator of mediator complex recruitment to nuclear receptor transcription complexes. Molecular Cell.

[54] Pan X, Hobbs RP, Coulombe PA (2013). The expanding significance of keratin intermediate filaments in normal and diseased epithelia. Current Opinion in Cell Biology.

[55] Wang W, Zhang W, Han Y, Chen J, Wang Y, Zhang Z, Hui R (2005). NELIN, a new F-actin associated protein, stimulates HeLa cell migration and adhesion. Biochemical and Biophysical Research Communications.

[56] He SJ, Cheng J, Feng X, Yu Y, Tian L, Huang Q (2017). The dual role and therapeutic potential of high-mobility group box 1 in cancer. Oncotarget.

[57] Vizcaino JA, Csordas A, Del-Toro N, Dianes JA, Griss J, Lavidas I, Mayer G, Perez-Riverol Y, Reisinger F, Ternent T, Xu QW, Wang R, Hermjakob H (2016). 2016 update of the PRIDE database and its related tools. Nucleic Acids Research.

[58] Fan J, Liu J, Culty M, Papadopoulos V (2010). Acyl-coenzyme A binding domain containing 3 (ACBD3; PAP7; GCP60): an emerging signaling molecule. Progress in Lipid Research.

[59] McGee AM, Douglas DL, Liang Y, Hyder SM, Baines CP (2011). The mitochondrial protein C1qbp promotes cell proliferation, migration and resistance to cell death. Cell Cycle.

[60] Scully OJ, Yu Y, Salim A, Thike AA, Yip GW, Baeg GH, Tan PH, Matsumoto K, Bay BH (2015). Complement component 1, q subcomponent binding protein is a marker for proliferation in breast cancer. Experimental Biology and Medicine.

[61] Reinert LS, Shi B, Nandi S, Mazan-Mamczarz K, Vitolo M, Bachman KE, He H, Gartenhaus RB (2006). MCT-1 protein interacts with the cap complex and modulates messenger RNA translational profiles. Cancer Research.

[62] Takahashi K, Suzuki K (2011). WAVE2, N-WASP, and Mena facilitate cell invasion via phosphatidylinositol 3-kinase-dependent local accumulation of actin filaments. Journal of Cellular Biochemistry.

[63] Santiago-Medina M, Yang J (2016). MENA Promotes Tumor-Intrinsic Metastasis through ECM Remodeling and Haptotaxis. Cancer Discovery.

[64] Cools J, DeAngelo DJ, Gotlib J, Stover EH, Legare RD, Cortes J, Kutok J, Clark J, Galinsky I, Griffin JD, Cross NC, Tefferi A, Malone J (2003). A tyrosine kinase created by fusion of the PDGFRA and FIP1L1 genes as a therapeutic target of imatinib in idiopathic hypereosinophilic syndrome. The New England Journal of Medicine.

[65] Iwasaki J, Kondo T, Darmanin S, Ibata M, Onozawa M, Hashimoto D, Sakamoto N, Teshima T (2014). FIP1L1 presence in FIP1L1-RARA or FIP1L1-PDGFRA differentially contributes to the pathogenesis of distinct types of leukemia. Annals of Hematology.

[66] Teng Y, Ai Z, Wang Y, Wang J, Luo L (2013). Proteomic identification of PKM2 and HSPA5 as potential biomarkers for predicting high-risk endometrial carcinoma. The Journal of Obstetrics and Gynaecology Research.

[67] Luo X, Yao J, Nie P, Yang Z, Feng H, Chen P, Shi X, Zou Z (2016). FOXM1 promotes invasion and migration of colorectal cancer cells partially dependent on HSPA5 transactivation. Oncotarget.

[68] Suetens A, Moreels M, Quintens R, Chiriotti S, Tabury K, Michaux A, Gregoire V, Baatout S (2014). Carbon ion irradiation of the human prostate cancer cell line PC3: a whole genome microarray study. International Journal of Oncology.

[69] Salza R, Peysselon F, Chautard E, Faye C, Moschcovich L, Weiss T, Perrin-Cocon L, Lotteau V, Kessler E, Ricard-Blum S (2014). Extended interaction network of procollagen C-proteinase enhancer-1 in the extracellular matrix. The Biochemical Journal.

[70] Li C, Ge M, Yin Y, Luo M, Chen D (2012). Silencing expression of ribosomal protein L26 and L29 by RNA interfering inhibits proliferation of human pancreatic cancer PANC-1 cells. Molecular and Cellular Biochemistry.

[71] Daftuar L, Zhu Y, Jacq X, Prives C (2013). Ribosomal proteins RPL37, RPS15 and RPS20 regulate the Mdm2-p53-MdmX network. PloS One.

[72] Sechler M, Borowicz S, Van Scoyk M, Avasarala S, Zerayesus S, Edwards MG, Kumar Karuppusamy Rathinam M, Zhao X, Wu PY, Tang K, Bikkavilli RK, Winn RA (2015). Novel Role for gamma-Catenin in the Regulation of Cancer Cell Migration via the Induction of Hepatocyte Growth Factor Activator Inhibitor Type 1 (HAI-1). The Journal of Biological Chemistry.

[73] Hu C, Jiang N, Wang G, Zheng J, Yang W, Yang J (2014). Expression of hepatocyte growth factor activator inhibitor-1 (HAI-1) gene in prostate cancer: clinical and biological significance. J BUON.

[74] Schwarz SE, Matuschewski K, Liakopoulos D, Scheffner M, Jentsch S (1998). The ubiquitin-like proteins SMT3 and SUMO-1 are conjugated by the UBC9 E2 enzyme. Proceedings of the National Academy of Sciences of the United States of America.

[75] Zhu S, Sachdeva M, Wu F, Lu Z, Mo YY (2010). Ubc9 promotes breast cell invasion and metastasis in a sumoylation-independent manner. Oncogene.

[76] Moschos SJ, Jukic DM, Athanassiou C, Bhargava R, Dacic S, Wang X, Kuan SF, Fayewicz SL, Galambos C, Acquafondata M, Dhir R, Becker D (2010). Expression analysis of Ubc9, the single small ubiquitin-like modifier (SUMO) E2 conjugating enzyme, in normal and malignant tissues. Human Pathology.

[77] Li H, Niu H, Peng Y, Wang J, He P (2013). Ubc9 promotes invasion and metastasis of lung cancer cells. Oncology Reports.

[78] Xu J, Footman A, Qin Y, Aysola K, Black S, Reddy V, Singh K, Grizzle W, You S, Moellering D, Reddy ES, Fu Y, Rao VN (2016). BRCA1 Mutation Leads to Deregulated Ubc9 Levels which Triggers Proliferation and Migration of Patient-Derived High Grade Serous Ovarian Cancer and Triple Negative Breast Cancer Cells. International Journal of Chronic Diseases and Therapy.

[79] De Marco M, Basile A, Iorio V, Festa M, Falco A, Ranieri B, Pascale M, Sala G, Remondelli P, Capunzo M, Firpo MA, Pezzilli R, Marzullo L (2017 Aug 31). Role of BAG3 in cancer progression: A therapeutic opportunity. Seminars in Cell and Developmental Biology.

[80] Xiao H, Cheng S, Tong R, Lv Z, Ding C, Du C, Xie H, Zhou L, Wu J, Zheng S (2014). BAG3 regulates epithelial-mesenchymal transition and angiogenesis in human hepatocellular carcinoma. Laboratory Investigation.

[81] Oliver D, Ji H, Liu P, Gasparian A, Gardiner E, Lee S, Zenteno A, Perinskaya LO, Chen M, Buckhaults P, Broude E, Wyatt MD, Valafar H (2017). Identification of novel cancer therapeutic targets using a designed and pooled shRNA library screen. Scientific Reports.

[82] Kobayashi H, Nishimura H, Matsumoto K, Yoshida M (2015). Identification of the determinants of 2-deoxyglucose sensitivity in cancer cells by shRNA library screening. Biochemical and Biophysical Research Communications.

[83] Chang JT, Wang F, Chapin W, Huang RS (2016). Identification of MicroRNAs as Breast Cancer Prognosis Markers through the Cancer Genome Atlas. PloS One.

[84] Zanini IM, Soneson C, Lorenzi LE, Azzalin CM (2017). Human cactin interacts with DHX8 and SRRM2 to assure efficient pre-mRNA splicing and sister chromatid cohesion. Journal of Cell Science.

[85] Suzuki M, Watanabe M, Nakamaru Y, Takagi D, Takahashi H, Fukuda S, Hatakeyama S (2016). TRIM39 negatively regulates the NFkappaB-mediated signaling pathway through stabilization of Cactin. Cellular and Molecular Life Sciences.

[86] Sturm D, Witt H, Hovestadt V, Khuong-Quang DA, Jones DT, Konermann C, Pfaff E, Tonjes M, Sill M, Bender S, Kool M, Zapatka M, Becker N (2012). Hotspot mutations in H3F3A and IDH1 define distinct epigenetic and biological subgroups of glioblastoma. Cancer Cell.

[87] Park SM, Choi EY, Bae M, Kim S, Park JB, Yoo H, Choi JK, Kim YJ, Lee SH, Kim IH (2016). Histone variant H3F3A promotes lung cancer cell migration through intronic regulation. Nature Communications.

[88] Courilleau D, Chastre E, Sabbah M, Redeuilh G, Atfi A, Mester J (2000). B-ind1, a novel mediator of Rac1 signaling cloned from sodium butyrate-treated fibroblasts. The Journal of Biological Chemistry.

[89] Mack NA, Whalley HJ, Castillo-Lluva S, Malliri A (2011). The diverse roles of Rac signaling in tumorigenesis. Cell Cycle.

[90] Cao WM, Murao K, Imachi H, Yu X, Abe H, Yamauchi A, Niimi M, Miyauchi A, Wong NC, Ishida T (2004). A mutant high-density lipoprotein receptor inhibits proliferation of human breast cancer cells. Cancer Research.

[91] Wei L, Xie X, Li J, Li R, Shen W, Duan S, Zhao R, Yang W, Liu Q, Fu Q, Qin Y (2015). Disruption of human vigilin impairs chromosome condensation and segregation. Cell Biology International.

[92] Yang WL, Wei L, Huang WQ, Li R, Shen WY, Liu JY, Xu JM, Li B, Qin Y (2014). Vigilin is overexpressed in hepatocellular carcinoma and is required for HCC cell proliferation and tumor growth. Oncology Reports.

[93] Zhou W, He MR, Jiao HL, He LQ, Deng DL, Cai JJ, Xiao ZY, Ye YP, Ding YQ, Liao WT, Liu SD (2015). The tumor-suppressor gene LZTS1 suppresses colorectal cancer proliferation through inhibition of the AKT-mTOR signaling pathway. Cancer Letters.

[94] Kpetemey M, Dasgupta S, Rajendiran S, Das S, Gibbs LD, Shetty P, Gryczynski Z, Vishwanatha JK (2015). MIEN1, a novel interactor of Annexin A2, promotes tumor cell migration by enhancing AnxA2 cell surface expression. Molecular Cancer.

[95] Schimmack S, Taylor A, Lawrence B, Alaimo D, Schmitz-Winnenthal H, Buchler MW, Modlin IM, Kidd M (2014). A mechanistic role for the chromatin modulator, NAP1L1, in pancreatic neuroendocrine neoplasm proliferation and metastases. Epigenetics and Chromatin.

[96] Gebhardt A, Habjan M, Benda C, Meiler A, Haas DA, Hein MY, Mann A, Mann M, Habermann B, Pichlmair A (2015). mRNA export through an additional cap-binding complex consisting of NCBP1 and NCBP3. Nature Communications.

[97] Narita T, Yung TM, Yamamoto J, Tsuboi Y, Tanabe H, Tanaka K, Yamaguchi Y, Handa H (2007). NELF interacts with CBC and participates in 3’ end processing of replication-dependent histone mRNAs. Molecular Cell.

[98] Liu S, Gao Y, Zhang C, Li H, Pan S, Wang X, Du S, Deng Z, Wang L, Song Z, Chen S (2016). SAMM50 Affects Mitochondrial Morphology through the Association of Drp1 in Mammalian Cells. FEBS Letters.

[99] Seong HA, Jung H, Manoharan R, Ha H (2011). Positive regulation of apoptosis signal-regulating kinase 1 signaling by ZPR9 protein, a zinc finger protein. The Journal of Biological Chemistry.

[100] Hu R, Peng G, Dai H, Breuer EK, Stemke-Hale K, Li K, Gonzalez-Angulo AM, Mills GB, Lin SY (2011). ZNF668 functions as a tumor suppressor by regulating p53 stability and function in breast cancer. Cancer Research.

[101] Deng SS, Xing TY, Zhou HY, Xiong RH, Lu YG, Wen B, Liu SQ, Yang HJ (2006). Comparative proteome analysis of breast cancer and adjacent normal breast tissues in human. Genomics, Proteomics and Bioinformatics.

