# Supplementary material for: Delineating the HMGB1 and HMGB2 interactome in prostate and ovary epithelial cells and its relationship with cancer
Source: Oncotarget. 2018 Apr 10;9(27):19050–64. doi: 10.18632/oncotarget.24887 (PMC5922377; doi:10.18632/oncotarget.24887)
Supplement: Supplementary file 1 [file oncotarget-09-19050-s001.pdf]

Delineating the HMGB1 and HMGB2 interactome in prostate and ovary epithelial cells and its relationship with cancer

SUPPLEMENTARY MATERIALS

Supplementary Dataset 1: PNT2 MS Data Analysis of HMGB1 co-immunoprecipitans. See Supplementary_Dataset_1
